# Transcriptomic analysis of neuregulin-1 regulated genes following ischemic stroke by computational identification of promoter binding sites: A role for the ETS-1 transcription factor

**DOI:** 10.1371/journal.pone.0197092

**Published:** 2018-06-01

**Authors:** Monique C. Surles-Zeigler, Yonggang Li, Timothy J. Distel, Hakeem Omotayo, Shaokui Ge, Byron D. Ford

**Affiliations:** 1 Department of Neurobiology, Morehouse School of Medicine, Atlanta, Georgia, United States of America; 2 Department of Biomedical Sciences, University of California–Riverside School of Medicine, Riverside, California, United States of America; 3 ICF, Atlanta, GA, United States of America; University of Kansas, UNITED STATES

## Abstract

Ischemic stroke is a major cause of mortality in the United States. We previously showed that neuregulin-1 (NRG1) was neuroprotective in rat models of ischemic stroke. We used gene expression profiling to understand the early cellular and molecular mechanisms of NRG1’s effects after the induction of ischemia. Ischemic stroke was induced by middle cerebral artery occlusion (MCAO). Rats were allocated to 3 groups: (1) control, (2) MCAO and (3) MCAO + NRG1. Cortical brain tissues were collected three hours following MCAO and NRG1 treatment and subjected to microarray analysis. Data and statistical analyses were performed using R/Bioconductor platform alongside Genesis, Ingenuity Pathway Analysis and Enrichr software packages. There were 2693 genes differentially regulated following ischemia and NRG1 treatment. These genes were organized by expression patterns into clusters using a K-means clustering algorithm. We further analyzed genes in clusters where ischemia altered gene expression, which was reversed by NRG1 (clusters 4 and 10). NRG1, IRS1, OPA3, and POU6F1 were central linking (node) genes in cluster 4. Conserved Transcription Factor Binding Site Finder (CONFAC) identified ETS-1 as a potential transcriptional regulator of NRG1 suppressed genes following ischemia. A transcription factor activity array showed that ETS-1 activity was increased 2-fold, 3 hours following ischemia and this activity was attenuated by NRG1. These findings reveal key early transcriptional mechanisms associated with neuroprotection by NRG1 in the ischemic penumbra.

## Introduction

Stroke is the 5^th^ leading cause of death in the United States, with ischemic stroke being most prevalent [1, 2]. The molecular events initiated by ischemic stroke activate several biological cascades, ultimately leading to cell death. Tissue plasminogen activator (tPA) is the only approved drug for treatment of ischemic stroke in the United States, but only 3–5% of patients fit the inclusion criteria for administration of this drug. Therefore, there is an urgent need to better understand the pathophysiology associated with ischemic stroke to reduce the associated cell death and develop more effective therapies.

Ischemic stroke occurs when the arterial blood supply to the brain is obstructed. Brain ischemia is characterized by an early onset neuronal death that begins within minutes following stroke [3–6]. This initial area of brain injury (the infarct core) occurs within minutes and is characterized by low cerebral blood flow, energy failure, and excitotoxicity. The resulting ischemic brain injury also is accompanied by increased synthesis of inflammatory molecules which causes damage to brain cells in a larger area of brain tissue which surrounds the necrotic core of the infarct (the ischemic penumbra) where the blood supply is compromised but not completely interrupted [7, 8]. In the penumbra, neurons can survive for several hours following stroke onset, suggesting that the therapeutic window for treating stroke can be quite prolonged. Studies have shown that the progression of damage in the ischemic core following ischemic stroke reaches its maximum size at around 3 hours [9, 10]. The progression of irreversible damage to the penumbra following stroke occurs within 3–24 hours after injury [9–14].

Neuregulins are a family of structurally related proteins that have diverse functions in the nervous system that have shown promise in treating stroke. Neuregulin 1 (NRG1) was discovered by multiple laboratories investigating cancer biology mechanisms, neuromuscular junction function and Schwann cell proliferation [15–20]. Many reports from our laboratory and others have shown that administration of NRG1 reduces neuronal damage and pro-inflammatory gene expression following middle cerebral artery occlusion (MCAO) in rats with an extended therapeutic window [21–28]. However, the transcriptional regulation of these processes is not completely understood. The identification of genes affected by NRG-1 treatment and the transcription regulators modulating these genes will provide important insight into the mechanism of NRG-1 protection following stroke.

Transcription factors (TF) are proteins that bind to regions of DNA sequences (transcription factor binding sites; TFBS) and control the expression of genes. The presence of specific TFBS in the regulatory regions of genes allows for the prediction of TF involved in the particular gene expression events. While several studies have used high-throughput analysis to examine gene expression changes after ischemic stroke, only a couple have used computational bioinformatics tools to predict transcriptional regulators that modulate the differentially expressed sets of genes [29, 30]. In this study, we performed a computational bioinformatics analysis to identify gene expression profiles and associated transcriptional mechanisms altered following NRG1 treatment at three hours following permanent MCAO using the CONserved transcription FACtor binding site finder (CONFAC) program [31]. CONFAC software allows for the computational prediction of TF that regulate genes of interest in a specific dataset. To capture the mechanisms involved in the initial stages of neuronal injury that occurs in the penumbra, we identified gene expression profiles altered following NRG1 treatment at 3 hours following ischemic stroke and the transcription factors that regulated the expression of these genes. Understanding these cellular and molecular mechanisms may give insight into mechanisms occurring in the ischemic penumbra and how neurons are protected by NRG1. These findings could support the development of clinical studies using NRG1 for the treatment of patients with acute stroke.

## Materials and methods

All animals were treated humanely and with regard for alleviation of suffering and pain and all surgical protocols involving animals were performed by sterile/aseptic techniques and were approved by the Institutional Animal Care and Use Committee at Morehouse School of Medicine prior to the initiation of experimentation. Male adult Sprague—Dawley *rattus norvegicus* (250-300g; Charles River Laboratory International, Inc., USA) were housed in standard cages in a temperature-controlled room (22 ± 2°C) on a 12 hour reverse light-dark cycle. Food and water were provided *ad libitum*.

Animals were randomly allocated into 3 groups: sham (control), MCAO + vehicle treatment (MCAO) and MCAO + NRG1 (MCAO+NRG1). Rats in the treatment groups (MCAO and MCAO+NRG1) were subjected to a left permanent MCAO. Rats were anesthetized with 5% isoflurane with an O_2_/N_2_O mixture (30%/70%) prior to surgery. After anesthesia administration, a rectal probe monitored the core body temperature, and a Homoeothermic Blanket Control Unit (Harvard Apparatus, Hollister, MA) was used to ensure the body temperature maintained at 37 degrees Celsius. Cerebral blood flow was monitored throughout the length of the surgery by a continuous laser Doppler flowmeter (Perimed, Ardmore, PA), with a laser Doppler probe placed 7 mm lateral and 2 mm posterior to bregma in a thinned cranial skull window.

MCAO was induced by the intraluminal suture method as we previously described [21]. Briefly, a 4 cm length 4–0 surgical monofilament nylon suture coated with silicon (Doccol Corp., Sharon, MA, diameter 0.37 mm, length 2.3–2.5 mm) was inserted from the external carotid artery (ECA) into the internal carotid artery (ICA) and then into the Circle of Willis, to occlude the origin of the left middle cerebral artery (MCA). Rats in the sham control group underwent the same procedure as those in the injury group, but a filament was not inserted into the ICA. Animals assigned into treatment groups (MCAO or MCAO+NRG1) were administered 50 μl of NRG1β reconstituted with 1% BSA in PBS (20 ug/kg; EGF-like domain, R&D Systems, Minneapolis, MN) or vehicle (1% BSA in PBS) All treatments were administered by bolus injection into the ICA through ECA immediately before MCAO, as previously described [32]. Animals were sacrificed three hours after MCAO. All NRG1 and vehicle treatment studies were performed in a blinded manner.

### Histology and immunohistology

A subset of the brains were used for 2,3,5-triphenyltetrazolium chloride (TTC) staining as previously described [33] (n = 3). With TTC labeling, the infarcted region appears white, whereas the normal non-infarcted tissue appears red. The remaining rats were anesthetized with 5% isoflurane and received saline followed by cold 4% PFA solution in PBS via transcardial perfusion. Brains were quickly removed and cryoprotected in 30% sucrose and cryosectioned at 20 μm thickness from the entire brain of each animal. Sections were mounted on slides which were stored at −80°C until further processed. Fluoro-Jade B (FJB, AG310, Millipore) labeling was performed as previously described [34]. To obtain FJB labeling, tissue sections were first dried at 50°C for 30 minutes, and 4% PFA was added to sections for 15 minutes. Sections were then washed with distilled water and incubated on a shaker table in 0.06% potassium permanganate (KMnO4) for 10 minutes and distilled water for 2 minutes. Fresh FJB solution (0.0004%) was then added to all sections for 20 minutes at room temperature. Sections were then rinsed in distilled ware dried at 50°C, cleared by immersion in xylene for 2 minutes and cover slipped with DPX mounting medium.

To quantify FJB labeling, every tenth section obtained from coronal sectioning of the forebrain of each rat (n = 3 for each condition) was labeled. A Nikon microscope equipped with CCD camera (Nikon, Melville, NY) was used to capture digital images of 2 sections in the piriform cortex at the same level across rats (at -0.30 mm from bregma) at 200X magnification. The number of FJB-positive cells was determined using Image J software and its automated cell counting feature (https://imagej.nih.gov/ij/; National Institutes of Health, Bethesda, MD). Only profiles of neuronal somas were counted and FJB-positive fragments were excluded. A mean value of FJB-positive cells per unit area within each brain region was obtained for each individual rat. These mean values from each rat were used as the statistical unit of measure for analysis by one-way ANOVA to determine statistically significant treatment effects.

### Microarray analysis

All animals were sacrificed three hours following MCAO (n = 3 for each condition) or sham surgery (n = 4). Brains were extracted and sectioned into 2 mm coronal sections (approximately +3.0 to −5.0 from bregma) using a brain matrix. The brains were separated at midline and the injured (left) cortical tissue was isolated. A left hemi-cortical tissue from the sham was used as the control. Total RNA was extracted with TRIzol Reagent (Life Technologies Corp, Carlsbad, CA), quality controlled and quantified by Agilent 2100 Bioanalyzer (Agilent Technology, Santa, Clara, CA). Microarrays were completed according to manufacturing guidelines (Affymetrix Inc., Santa, Clara, CA), with cRNA hybridized to an Affymetrix Rat Genome 2.0st Gene Chip (Affymetrix Inc.). The chips were processed and scanned according to manufacturing guidelines. Microarray chips were used for each of experimental sample groups.

Statistical analysis of microarray data was completed using the Bioconductor/R platform [35]. The oligo package, Robust Multi-Array Average (RMA) algorithm, was used to normalize data [36]. The Limma software package was used to conduct a Bayes moderated t-statistics analysis for each gene within the three experimental groups to identify differentially expressed genes (p-value <0.05) [37]. In Limma, the pairwise t test is conducted then adjusts p value by the Benjamini-Hochberg (BH) correction method for false discovery rate (FDR). Genesis software (http://genome.tugraz.at/) was used to complete the k-means cluster analysis (k = 10, user defined). Data underlying the study are within the paper and its Supporting Information files (S1 Table), and expression analysis data are available at the NCBI Gene Expression Omnibus (GEO) Repository (GSE103661).

Genes within each cluster of interest were uploaded to Ingenuity Pathway Analysis (IPA) software (Qiagen, Redwood City, CA; www.ingenuity.com). The gene datasets were analyzed between April 15, 2016, and April 30, 2016, using IPA. Genes that were present in the Ingenuity Knowledge Base (manually curated data from journals and external databases) were deemed “analysis-ready” by IPA and further analyzed. The right-tailed Fisher Exact Test was used to calculate a p-value determining a function or pathway in the Global Functional Analysis and Global Canonical Pathway that is due to random chance. Network, biological relationships, and functional analyses were created from the Ingenuity Knowledge Base. IPA also enabled the identification of direct (physical interaction) and indirect (no physical interaction) biological relationships created by “growing” the network of analysis-ready gene. The networks were “grown” by adding 15 additional molecules (user defined), to capture interactions/relationships between the analysis-ready genes. The genes added to the network were selected via the Ingenuity Network Generation Algorithm. This algorithm facilitated the addition of genes with the most biological relationships with the analysis-ready genes (cluster genes), while limiting the relationships between these 15 genes. The node genes in a network are defined as those that are highly connected and may do a large amount of the overall regulation of other genes in the network.

Enrichr (http://amp.pharm.mssm.edu/Enrichr/) is a web-based user interface, which allows users upload a list of genes [38]. The database retrieves data from many biological databases, which allows for the identification of associated pathways and ontology tools, to name a few. In the database, gene ontology (GO) biological processes 2015 were used to detect the top 5 biological processes (unadjusted p-values) associated with genes within the identified clusters. The Enrichr adjusted p-value value was used to identify the most critical processes. Enrichr calculates this value by using a rank based ranking; they use the Fisher exact test on multiple random gene sets to calculate the mean rank and standard deviation from the expected rank for each term in the gene set library. The z-score is then computed to evaluate the deviation from the expected rank. The p-values and associated adjusted p-values are provided to the user.

A tab-delimited text file containing the gene name and RefSeq ID of the genes in the clusters of interest was uploaded to the CONFAC web browser interface (http://confac.emory.edu/). The dataset was analyzed between April 1, 2016, and May 15, 2016. Due to the genomic similarity between the two species, mouse and rats, (~90%) we placed the list of rat genes into CONFAC). CONFAC software identified mouse orthologs from the uploaded gene list (from UCSC and ENSEMBL genomes). The software uses the UCSC genome database to identify 3kB genomic sequence in the proximal promoter region. CONFAC software next identified significantly conserved sequences (e-value < 0.001), which were analyzed for TFBS using MATCH™ software. TFBS and associated position weight matrices (PWM) were identified for genes that were conserved following a MATCH analysis with a matrix similarity of 0.85 and core similarity of 0.95. The final output table, which consisted of the cohort list of genes and the position weight matrix (collected in TRANSFAC® database), identified potential TFBS and the number of TFBS determined for each gene of interest. The CONFAC program then allowed for additional analysis using a Mann-Whitney U-test, which facilitated the statistical analysis of TFBS over-represented in the sample dataset compared to the 17 control random control datasets provided by CONFAC [31]. A TFBS was determined to be of over-represented if it was significantly increased following Mann-Whitney U tests against all 17 control datasets (p = 0.05). Enrichr (http://amp.pharm.mssm.edu/Enrichr/) is a web-based user interface, which allows users upload a list of molecules and the database retrieves data from many biological databases allowing for the identification of associated pathways and ontology tools [38, 39]. Gene ontology (GO) was used to detect biological processes associated with the CONFAC predicted TF. The top 5 most significant (unadjusted p-values) biologically processes were selected for this analysis. The Enrichr adjusted p-value value was used to identify the most critical processes. Enrichr calculates this value by using a rank based ranking; they use the Fisher exact test on multiple random gene sets to calculate the mean rank and standard deviation from the expected rank for each term in the gene set library. The z-score is then computed to evaluate the deviation from the expected rank. The p-values and associated adjusted p-values are provided to the user.

The Search Tool for the Retrieval of Interacting Genes/Proteins (STRING) database (http://string-db.org) was used to analyze the protein—protein interactions between the CONFAC predicted TF [40]. The protein-protein interactions are based on both known and predicted interactions, with line thickness indicating the strength of data support. STRING analysis determining the confidence of interaction (line thickness) by collecting data from multiple databases to determine the interaction likelihood. Confidence score are calculated by combining the probabilities from each evidence category including, but not limited to, prior co-expression, experimental data, association in curated databases and co-mentioned in Pub-Med abstracts.

### Real-time RT-PCR

Total RNA was extracted from tissue in each group (control, MCAO, and MCAO+NRG1) and quantified, as stated above. Quantitative real-time PCR (qRT-PCR) was performed using the iTaq Universal SYBR Green One-Step Kit (Bio-Rad Laboratories, Inc., Hercules, California) along with custom oligo primers for IRS1 (forward: CTGCATCGGACTCTACCAGG; reverse: CGAGGACTAAGTCTCCCCCA), OPA3 (forward: AGAGTGCAATTGATGCCCCA; reverse: TGAGGGGATCTGTACAGGCA) POU6F1 (forward: TGACTATGCTCTTCCTCCCCT; reverse: CACAGGAGGGGAAATACAGTTGA); SK1/Kcnn1 (forward: TCATCTCCATTACCTTCCTG; reverse: AGCCTGGTGTGTTTGTAGAT), SK2/Kcnn2 (forward: ACCTGCACGAGATGGACTCA; reverse: TTGTGCTCCGGCTTAGACAC); ERG1 (forward: CAACATCGCCACAGGAGA; reverse: ACACGGTACTCAGGGTCCAT) ERG2/Kcnh6/Kv11.2 (forward: AGATTGGAGTCCCGTGTGTC; reverse: TCCCACCAGAAGCGTAGACT) (Life Technologies, Rockville, MD), according to manufacturing guidelines. Reactions were carried out using a Bio-Rad CFX96™ Real-Time System mounted on a C1000™ Thermal Cycler with a reaction volume of 10ul. The average ΔCt was calculated for each experimental group (n = 3 for each condition), where ΔCt = Ct (gene of interest)–Ct (Housekeeper gene–GAPDH).

### Multiplex transcription factor assay

Brain tissues from the ipsilateral cortex, lateral to sections obtained for RNA extractions, was used for these studies (n = 3 for each condition). Nuclear proteins were isolated using the USB Nuclear Extraction Kit (Affymetrix, Inc.). The Procarta Transcription Factor Assay (Affymetrix, Inc.) was used to measure transcription factor DNA binding activity. The Procarta transcription factor DNA detection probes and nuclear extracts from samples were incubated for 30 minutes at 15°C to form a protein-DNA complex. Procarta controls (n = 3) and blanks (n = 3) samples were also added. Unbound protein and DNA were removed by adding a cold binding buffer to samples on a 96-well filtered plate (separation plate) and placement of a collection plate below. The samples were washed, incubated on ice for 5 minutes (1x), centrifuged for 5 minutes at 4°C, and flow through discarded. Each step was repeated five times and centrifuged for 3 minutes at 4°C. Protein-DNA complex were denatured at 95°C for 5 minutes, and then incubated with capture beads for 30 minutes at 50°C on a shaker for 400 rpm. Capture beads were washed, and Streptavidin-PE was added to for 30 minutes at room temperature on a shaker for 400 rpm. Capture beads are then washed and resuspended in reading buffer on a shaker at 400 rpm and read on the Luminex instrument (Bio-plex system 200, Bio-Rad, Hercules, California). All protocols were performed according to the manufacturer’s guidelines.

## Results

**Fig 1:**
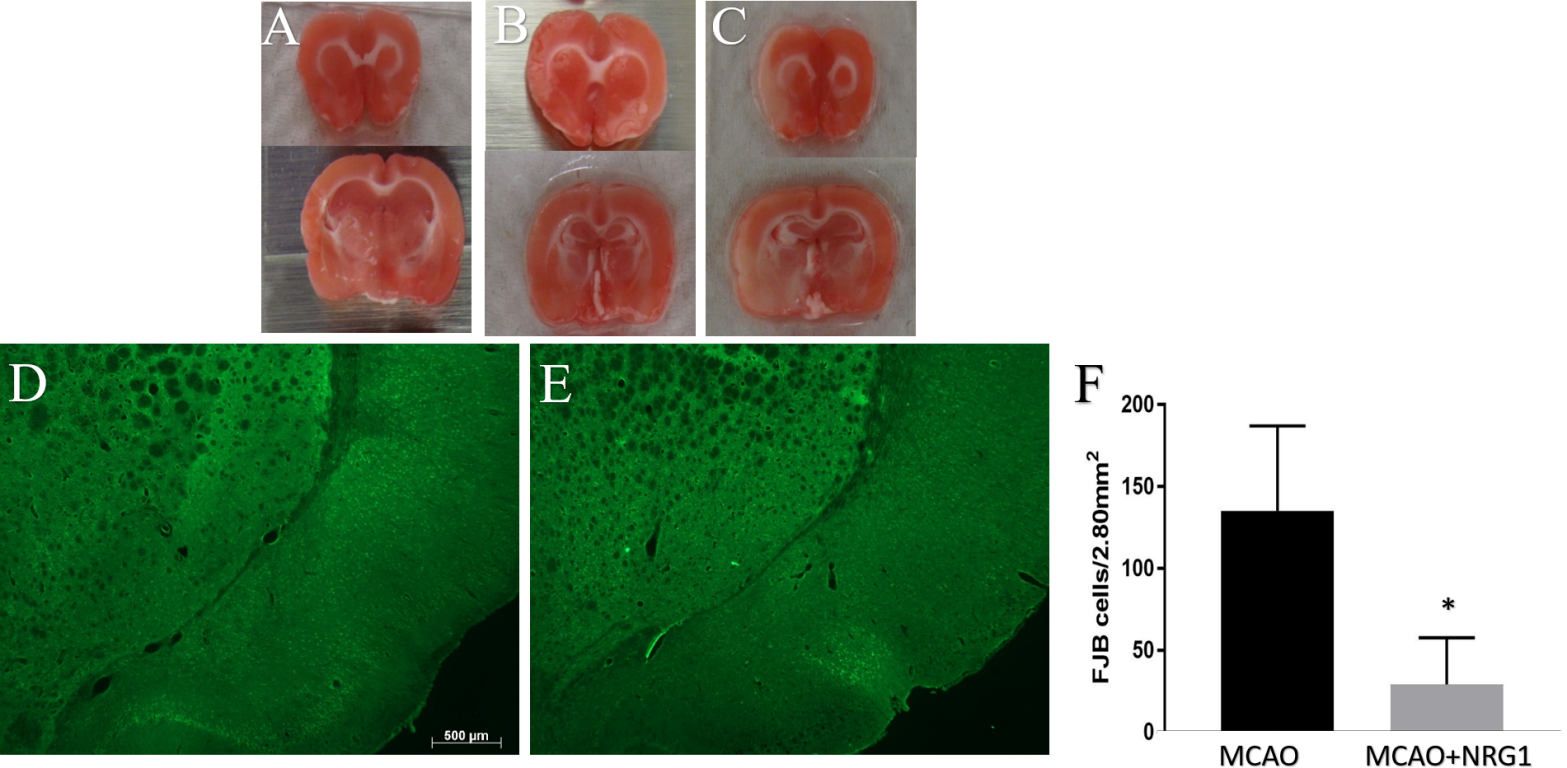
Cell markers of injury following permanent MCAO. There was no obvious difference in TTC staining in either (A) MCAO or (B) MCAO + NRG1 tissue 3 hours after injury compared to damage 24 hours after MCAO (C). At 3 hours following MCAO, FJB labeled degenerating/injured cells were detected in the injured cortex following vehicle treatment (D), which were dramatically reduced following NRG1 treatment (E,F). Graph indicates mean value ± SEM; n = 4; p < 0.05.

We previously demonstrated neuroprotection by NRG1 following ischemia in rat models [32, 33, 41–43]. Using MRI, we showed that the neuroprotective effects were seen as early as 1 hour following ischemia in subcortical brain regions [33]. In these studies, rats were treated with NRG1 immediately before MCAO for 3 hours and showed that the neuronal injury was limited to subcortical regions at 3 hours post MCAO and later spread to the cortex, indicative of an ischemic penumbra [33]. Therefore, we examined gene expression profiles in the ischemic brain cortex following vehicle and NRG-1 treatment. Minimal TTC staining is detected in brain tissues 3 hours following MCAO (Fig 1A) or MCAO + NRG1 treatment (Fig 1B) compared to damage after 24 hours (Fig 1C). Fluoro JadeB (FJB) labeled neurons primarily in the striatum but limited to the piriform cortex 3 hours after MCAO, indicating early neuronal injury in the ischemic penumbra (Fig 1D). The presence of FJB labeled cells was decreased by 80% in the cortex of animals treated with NRG1 (Fig 1E and 1F).

**Fig 2:**
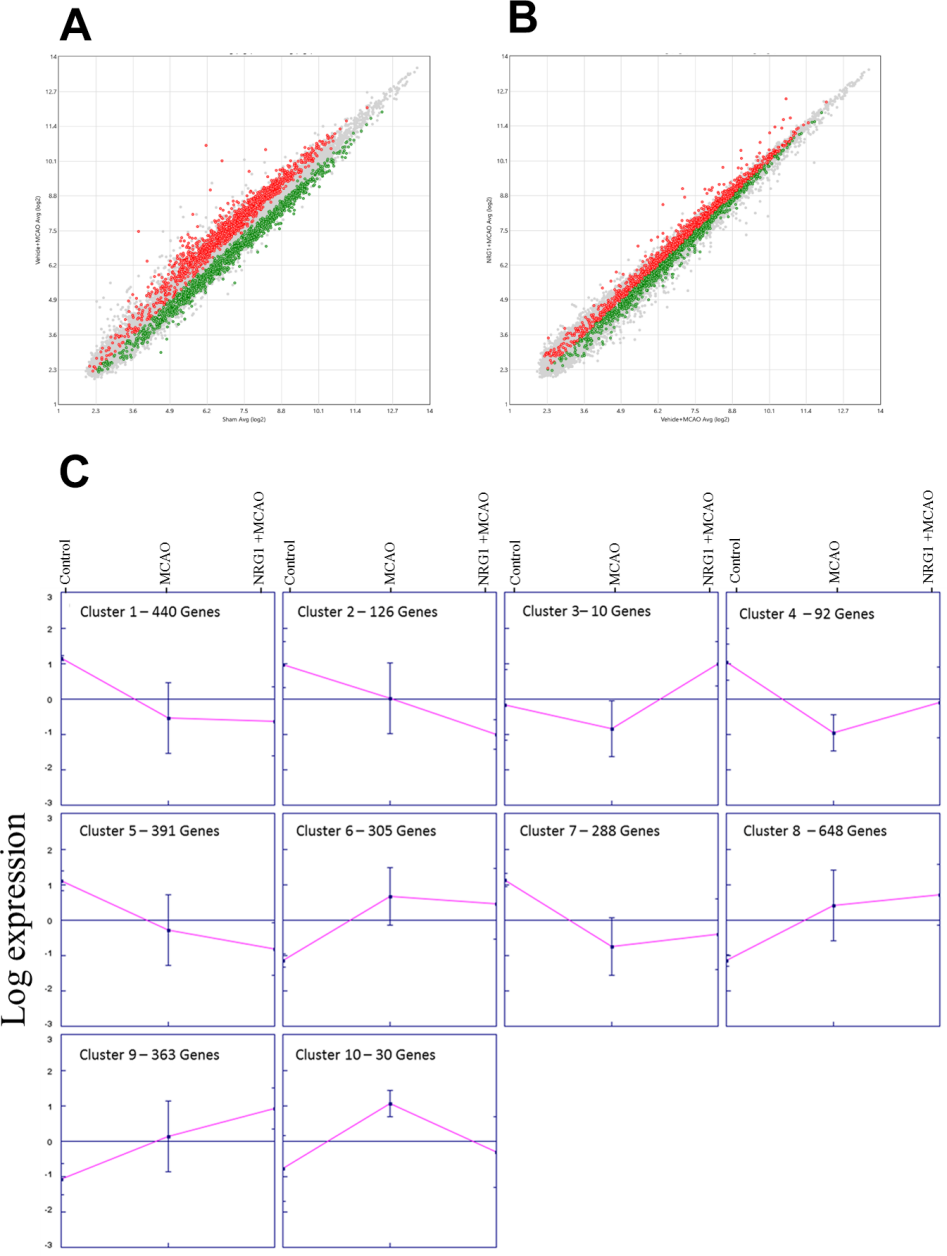
The MA plots show the correlation of the hybridization signal intensity for the expressed genes comparing shams to MCAO (A) and MCAO + either vehicle or NRG-1 treatment (B). In cluster analysis expression profiles (C), K means cluster algorithm (k = 10) was used to identify gene expression among statistically significant genes (ANOVA, p < 0.05) profiles in sham, MCAO and MCAO+NRG1 treated animals at 3 hours following injury. Within each cluster illustration, the grey line represents the mean gene expression values for each gene in the cluster, while the pink line represents the mean expression profile within each cluster.

To examine how NRG1 alters early gene expression in the ischemic penumbra following brain ischemia, microarray analysis was performed on cortical tissues from control, MCAO, and MCAO+NRG1 animals three hours following MCAO. Of the 36,685 gene probes on the Affymetrix rat 2.0st chip, 19,866 genes were annotated for the rat genome. The MA plots show the relationship between the signal intensity for the expressed genes and difference between two conditions for those genes. The plots in Fig 2A compare the gene intensities of the genes present in the experiments for the sham control and MCAO. Following MCAO, there was a dramatic increase in the number of genes upregulated and downregulated. When we compared experiments from MCAO and animals treated with NRG-1 before MCAO (Fig 2B), there were many genes which were up- and downregulated by NRG-1. R/Bioconductor analysis indicated that 2,693 genes were differentially expressed among the three treatment groups (p<0.05). These genes were selected for further analysis. The Genesis software (http://genome.tugraz.at/) k-means clustering algorithm was used to identify genes with similar mean expression patterns across all experimental groups and presented 10 expression profiles (Fig 2C). For each cluster, the baseline mean value for the genes was compared to levels following MCAO and to MCAO with NRG1 treatment. The genes located in each cluster are listed in S1 Table. We focused on genes within clusters whose mean expression was altered following MCAO and trended toward baseline expression following NRG1 treatment. Clusters 3, 4 and 10 all fit this selection criterion and were selected as our clusters of interest. Cluster 3 was excluded from further analysis due to the limited number of genes in this cluster to analyze (n = 10). Unique gene ontology terms are associated with each expression clustering profile, providing information about the biological processes associated with genes altered following MCAO and/or NRG1 (Table 1). Enricher software was used to identify gene ontology terms associated with this dataset and on which all sequential analyses were completed.

**Table 1:** Gene ontology ID and biological processes associated with genes altered following stroke and NRG1.

Cluster	Top biological processes	Gene ontology ID	Gene (n)	P-values	Adjusted P-values	Genes
Cluster 1	neuron projection guidance	GO:0097485	24	1.64E-06	0.002121	ARHGEF11, VASP, SEMA6C, CNTNAP1, UNC5A, SRC, NTN4, GATA3, SEMA3F, GFRA3, MAPK8IP3, ARHGAP35, GLI2, ABLIM1, COL3A1, CACNB3, FGF8, DLG4, GPC1, PLA2G10, PLXNA1, COL6A3, PIP5K1C, AGRN
	axon guidance	GO:000741	24	1.64E-06	0.002121	ARHGEF11, VASP, SEMA6C, CNTNAP1, UNC5A, SRC, NTN4, GATA3, SEMA3F, GFRA3, MAPK8IP3, ARHGAP35, GLI2, ABLIM1, COL3A1, CACNB3, FGF8, DLG4, GPC1, PLA2G10, PLXNA1, COL6A3, PIP5K1C, AGRN
	extracellular matrix organization	GO:0030198	22	1.19E-05	0.008006	RAMP2, PTPRS, VWF, ELN, SDC3, ICAM2, NTN4, LTBP4, FURIN, LTBP3, LOXL1, COL1A1, COL3A1, COL1A2, CDH1, MMP15, PECAM1, COL6A3, CAPN1, FMOD, AGRN, ENG
	extracellular structure organization	GO:0043062	22	1.24E-05	0.008006	RAMP2, PTPRS, VWF, ELN, SDC3, ICAM2, NTN4, LTBP4, FURIN, LTBP3, LOXL1, COL1A1, COL3A1, COL1A2, CDH1, MMP15, PECAM1, COL6A3, CAPN1, FMOD, AGRN, ENG
	response to transforming growth factor beta signaling	GO:0007179	12	4.48E-05	0.023244	NCOR2, TGFBR3, COL3A1, COL1A2, SRC, ID1, LTBP4, FURIN, LTBP3, TAB1, ENG, TGFBR2
Cluster 2	Wnt signaling pathway	GO:0016055	9	4.22E-06	0.004582	SLC9A3R1, TCF7L1, FZD2, JUP, TGFB1I1, KREMEN1, WNT9B, NLK, BCL9L
	canonical Wnt signaling pathway	GO:0060070	5	9.46E-05	0.035693	TCF7L1, FZD2, JUP, WNT9B, BCL9L
	tissue morphogenesis	GO:0048729	9	0.000109	0.035693	TCF7L1, JUP, HEY1, SOX18, TGFB1I1, WNT9B, SCRIB, SOX8, TBX18
	regulation of stem cell differentiation	GO:2000736	5	0.00135	0.035693	TCF7L1, JUP, TGFB1I1, CNOT3, BCL9L
	aorta morphogenesis	GO:0035909	3	0.00164	0.035693	PDGFRB, SEC24B, HEY1
Cluster 3	fucose catabolic process	GO:0019317	1	0.002101	0.014878	FUT4
	L-fucose metabolic process	GO:0042354	1	0.002101	0.014878	DPPA3
	L-fucose catabolic process	GO:0042355	1	0.002101	0.014878	FUT4
	regulation of DNA methylation	GO:0044030	1	0.003361	0.014878	FUT4
	fucose metabolic process	GO:0006004	1	0.003571	0.014878	FUT4
Cluster 4	synaptic transmission	GO:0007268	7	0.001418	0.310188	GRM2, KCNH3, CHRNB3, KCNH6, UNC119, KCNN1, KCNK16
	inorganic cation transmembrane transport	GO:0098662	7	0.001532	0.310188	KCNH3, CACNG7, JPH3, KCNH6, ORAI1, KCNN1, KCNK16
	potassium ion transmembrane transport	GO:0071805	4	0.001682	0.310188	KCNH3, KCNH6, KCNN1, KCNK16
	cellular potassium ion transport	GO:0071804	1	0.001682	0.310188	KCNH3, KCNH6, KCNN1, KCNK16
	regulation of smoothened signaling pathway	GO:0008589	3	0.002068	0.310188	EVC, SALL3, GLIS2
Cluster 5	eye morphogenesis	GO:0048592	6	0.000303	0.585461	RING1, LRP5, COL8A2, BMP7, GLI3, AQP1
	camera-type eye morphogenesis	GO:0048593	4	0.000493	0.585461	RING1, COL8A2, GLI3, AQP1
	positive regulation of angiogenesis	GO:0045766	8	0.000815	0.585461	CCBE1, NOS3, TNFSF12, CHI3L1, PRKD2, GATA2, AQP1, HDAC7
	regulation of dendrite development	GO:0050773	7	0.001783	0.585461	NUMBL, PALM, SARM1, BMP7, SRCIN1, LZTS1, DBN1
	fatty acid transport	GO:0015908	4	0.002872	0.585461	NMB, SLCO2A1, ACACB, ABCD1, SLC27A5
Cluster 6	negative regulation of fibroblast proliferation	GO:0048147	5	9.71E-05	0.176748	MYC, MED31, PMAIP1, EMD, CDC73
	skeletal muscle cell differentiation	GO:0035914	6	0.000149	0.176748	EGR1, NR4A1, EGR2, KLF5, CITED2, EMD
	histone monoubiquitination	GO:0010390	4	0.000606	0.356058	RYBP, CDC73, SKP1, CUL4B
	DNA-templated transcription, initiation	GO:0006352	11	0.000853	0.356058	NR4A1, NR4A3, TBP, CCNH, MYC, MED31, SMARCA5, UBE2D1, GTF2H2, JUNB, MED7
	negative regulation of bone resorption	GO:0045779	4	0.000895	0.356058	TNFAIP3, TNFRSF11B, VEGFA
Cluster 7	neuron projection morphogenesis	GO:0048812	12	1.13E-05	0.025439	NTRK1, BRSK1, AMIGO1, MINK1, DVL1, DAB2IP, BAI1, NPTX1, CELSR2, DACT1, PACSIN1, SHANK1
	cell projection morphogenesis	GO:0048858	12	0.000129	0.078063	NTRK1, BRSK1, AMIGO1, MINK1, DVL1, DAB2IP, BAI1, NPTX1, CELSR2, DACT1, SHANK1, PACSIN1
	regulation of ion transmembrane transport	GO:0034765	14	0.000192	0.078063	HCN3, GSTM2, KCNJ10, KCNJ12, PDE4D, KCNC4, MINK1, KCNAB2, CACNA1G, CACNA1I, CALHM1, HCN2, KCNH1, SHANK1
	inorganic cation transmembrane transport	GO:0098662	17	0.000209	0.078063	HCN3, SLC13A4, KCNJ10, SLC30A3, KCNJ12, KCNC4, ATP1A2, ATP2B2, KCNAB2, PKD1, CACNA1G, RHD, CACNA1I, CALHM1, GAS6, HCN2, KCNH1
	regulation of synaptic transmission	GO:0050804	12	0.000244	0.078063	NTRK1, NEUROD2, GRM4, KCNJ10, GRIK5, KCNC4, RARA, CSPG5, ATP2B2, NAT8
Cluster 8	regulation of RNA splicing	GO:0043484)	13	3.57E-06	0.00646	MBNL1, DDX5, RBM8A, CELF1, SRSF1, HNRNPLL, CLK4, PTBP2, CLK3, CLK1, HNRNPH1, SRSF10, SRSF12
	mRNA splice site selection	GO:0006376	8	4.57E-06	0.00646	SF3A3, MBNL1, CELF1, YTHDC1, SRSF1, SRSF10, PTBP2, SRSF12
	alcohol biosynthetic process	GO:0046165	15	6.6E-06	0.00646	IDI1, PRKAA2, SAMD8, GGPS1, CHKA, CNBP, INSIG1, MSMO1, HMGCR, HSD17B7, CYB5R1, IMPA1, ACER3, CHPT1, CEPT1
	regulation of translation	GO:0006417	25	7.8E-06	0.00646	EIF4A2, PPP1R15A, BTG2, DDX3X, RBM8A, CELF1, FMR1, EIF5A2, SRSF1, RBM4B, MTIF2, HRSP12, ZFP36L1, SYNCRIP, RGS2, ZFP36, POLR2G, EIF2S1, RHOA, CNOT6, EIF3M, IMPACT, CNOT7, CPEB3, SELT
	posttranscriptional regulation of gene expression	GO:0010608	34	1.09E-05	0.007249	EIF4A2, PPP1R15A, BTG2, DDX3X, RBM8A, CELF1, FMR1, EIF5A2, SRSF1, PTEN, RBM4B, MTIF2, HRSP12, ZFP36L1, SYNCRIP, RGS2, ZFP36, POLR2G, SIAH1, RC3H1, EIF2S1, RHOA, CNOT6, EIF3M, IMPACT, CNOT7, RNF149, MDM2, BCL2, NAA15, TARDBP, CPEB3, SELT, RBM24
Cluster 9	nuclear import	GO:0051170	8	7.59E-05	0.194226	RANBP2, NOP58, FGF9, PHIP, JAK2, KPNA3, HTATIP2, IPO5
	protein import into nucleus	GO:0006606	7	0.000334	0.284991	RANBP2, NOP58, FGF9, PHIP, JAK2, KPNA3, IPO5
	single-organism nuclear import	GO:1902593	7	0.000334	0.284991	RANBP2, NOP58, FGF9, PHIP, JAK2, KPNA3, IPO5
	protein localization to nucleus	GO:0034504	8	0.000503	0.321731	RANBP2, OSBPL8, NOP58, FGF9, PHIP, JAK2, KPNA3, IPO5
	negative regulation of transmembrane receptor protein serine/threonine kinase signaling pathway	GO:0090101	8	0.000719	0.335457	GREM1, PPP1CB, PPP1CC, CAV2, VWC2L, LEMD3, SKIL, UCHL5
Cluster 10	cellular response to interferon-alpha	GO:0035457	1	0.007542	0.139971	IFIT2
	microtubule depolymerization	GO:0007019	1	0.007542	0.139971	KIF2B
	negative regulation of mast cell activation	GO:0033004	1	0.007542	0.139971	MILR1
	behavioral response to nicotine	GO:0035095	1	0.007542	0.139971	CHRNB4
	leukocyte aggregation	GO:0070486	1	0.008377	0.139971	S100A8

**Fig 3:**
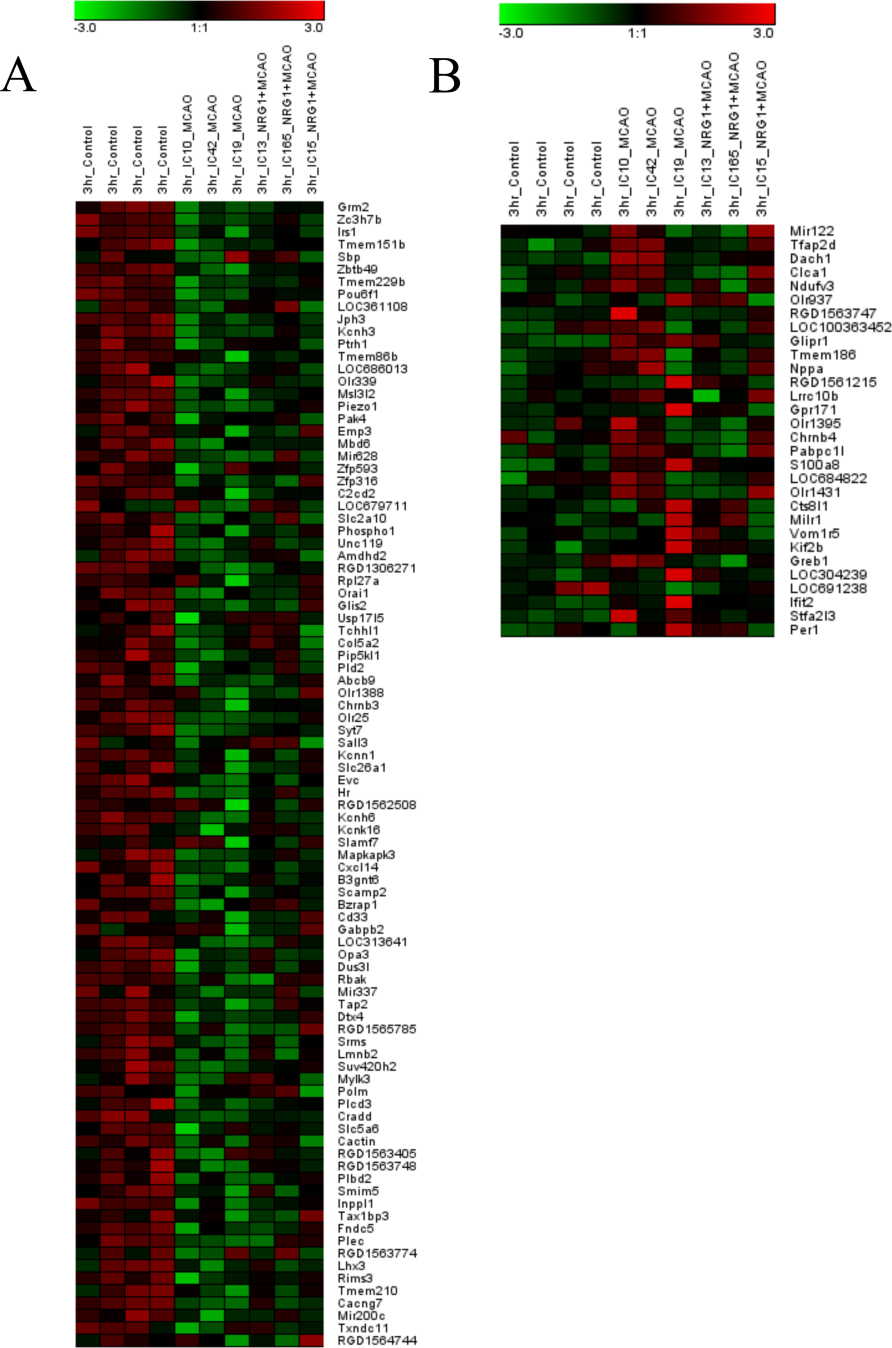
A heat map visualized the expression intensities for significant statistical genes in (a) cluster 4, decreased following MCAO and returned to baseline by NRG1, while genes in (b) cluster 10 had the opposite expression profile. High signal (red) to low signal (green) intensities.

**Fig 4:**
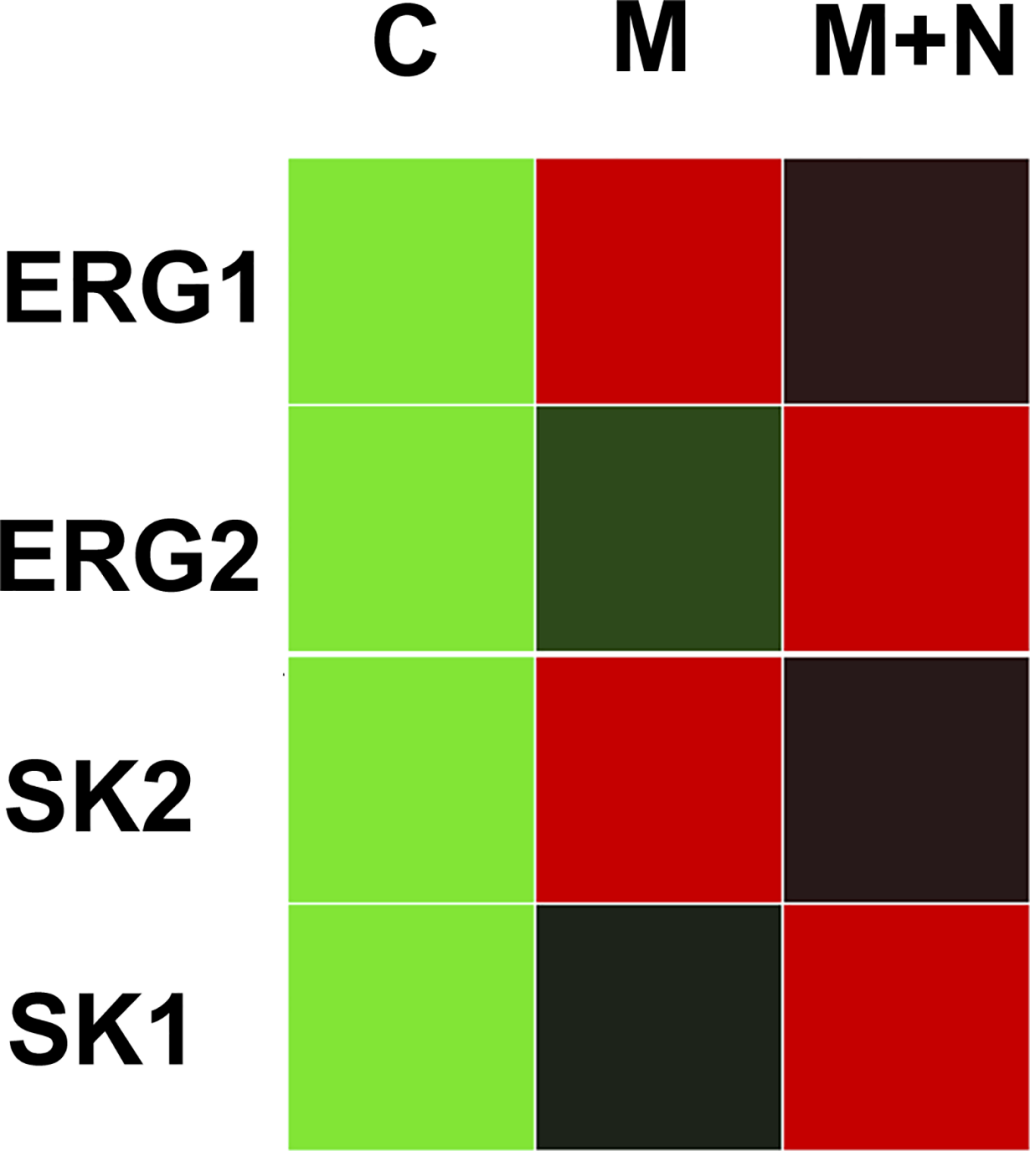
KCN Channel qPCR. ERG2 and SK1 both showed expression patterns by qPCR similar to the microarray analysis. ERG1 and SK2 channel homologues, which were not identified in the microarray analysis, showed different expression patterns (C = control; M = MCAO; M+N = MCAO+NRG1).

**Fig 5:**
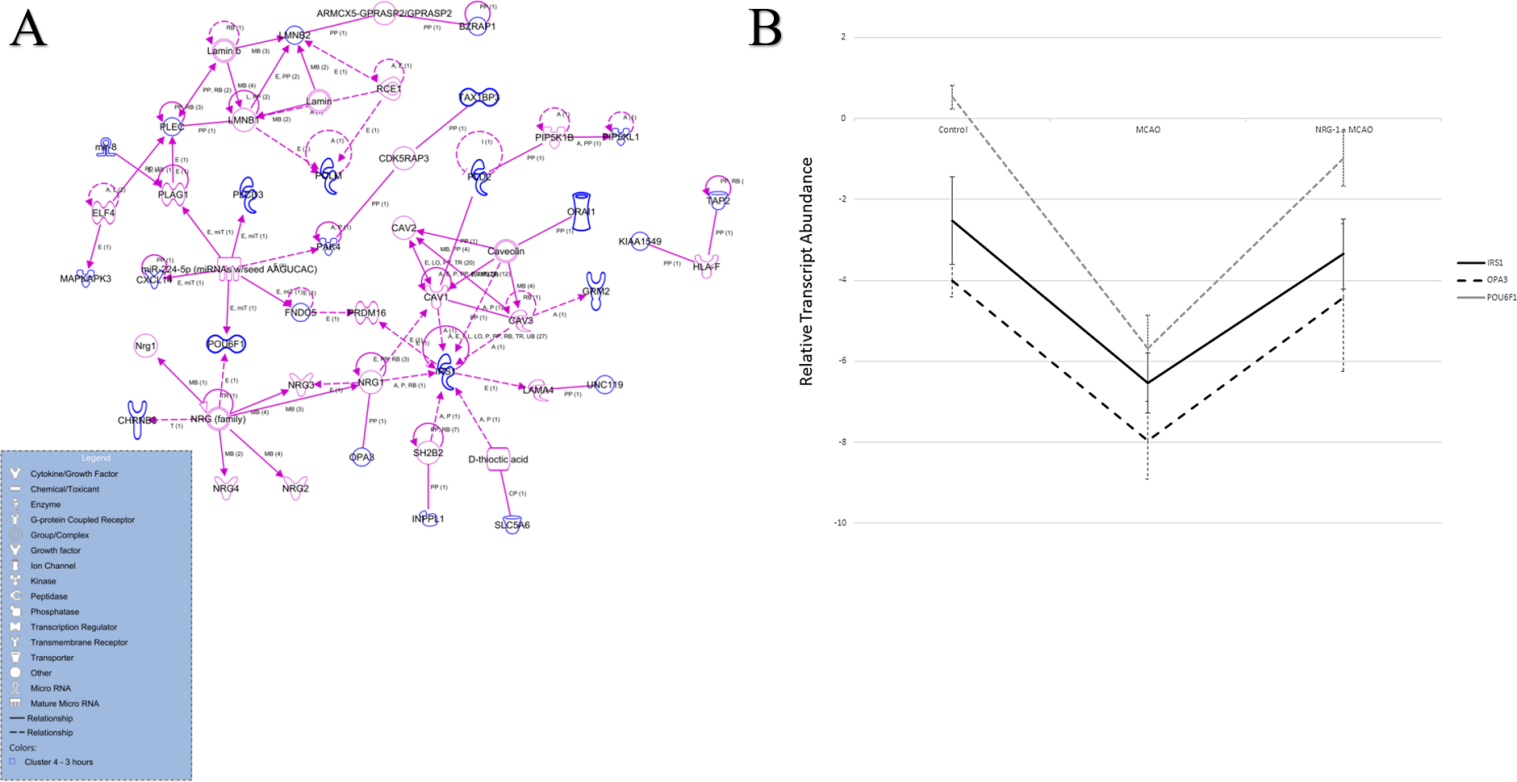
IPA Network Analysis Genes in Cluster 4. (a) IPA Network analysis was generated by seeding the genes within cluster 4 (blue highlighted genes) and “growing” those genes into a network by displaying direct (solid lines) and indirect (dashed lines) connections. All orphan genes were removed. Genes added to the network are illustrated with purple lines. (b) qRT-PCR was performed for node genes IRS1, OPA3, and POU6F1 to identify the associated gene expression profiles. The average ΔCt was calculated for each experimental group, where ΔCt = Ct (gene of interest)–Ct (Housekeeper gene–GAPDH). Data are shown ± S.D.

In cluster 4, ninety-two genes were reduced following MCAO and returned to near baseline levels by NRG1 treatment as indicated in the hierarchical cluster (Fig 3). The KCN gene family was well represented in the analysis, qPCR results confirmed the presence and expression pattern of 2 of these genes (Fig 4). ERG1 and SK2 both showed expression patterns by qPCR similar to the microarray analysis. ERG1 and SK1 channel homologues, which were not identified in the microarray analysis, showed different expression patterns. In addition, IPA identified 83 of the 92 genes as analysis-ready, and the biological relationships between these genes were visualized, and functional analysis was completed. Biological relationships were identified for 24 genes within the network, with NRG1 being one of the center linking nodes between these genes (Fig 5A). The genes of interest with connections to NRG1 included *OPA3*, *IRS1*, *CHRNB3* and *POU6F1*. We used qPCR to validate the expression patterns of genes altered by ischemia and NRG1. *IRS1*, *OPA3* and *POU6F1*, all behaved in the same pattern as seen in the microarray analysis (Fig 5B). In cluster 10, thirty genes increased following MCAO and trend toward baseline after NRG1 treatment.

**Fig 6:**
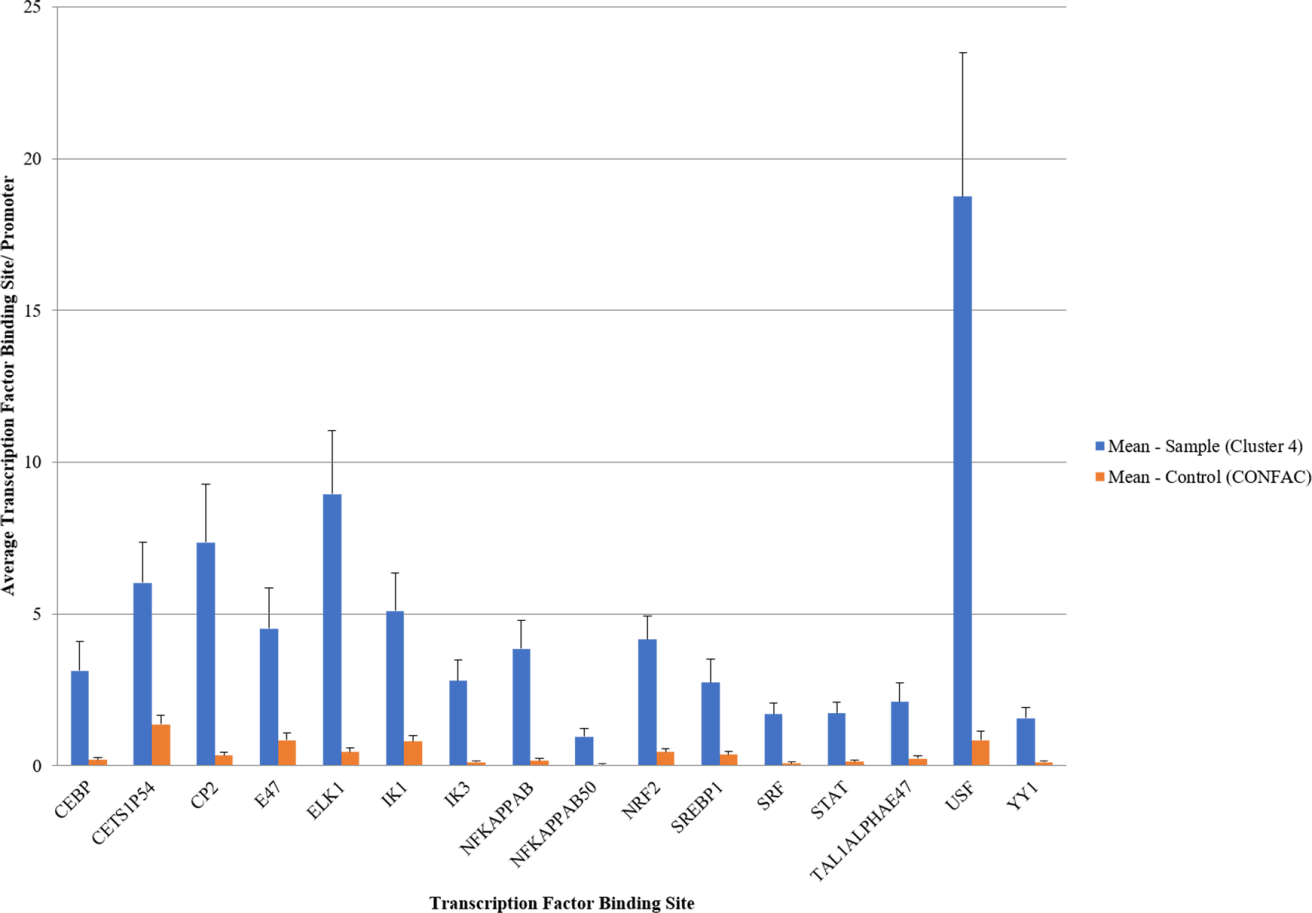
CONFAC database analysis. CONFAC identified the conserved transcription factor binding sites (TFBS) associated with genes in cluster 4 (per promoter). A statistical comparison using the Mann-Whitney U test identified statistically over-represented TFBS in the datasets when compared to the 17 randomized controls. There were 16 TFBS predicted to regulate gene expression profile of cluster 4.

CONFAC software (http://confac.emory.edu/) was used to predict transcription factors that could regulate the genes found in the k-means cluster analysis. CONFAC determines TFBS that are statistically overrepresented in a gene dataset compared to a random set of genes. Of the 92 genes identified in cluster 4, mouse orthologs were recognized for 52 genes. TFBS were identified for 39 of the 52 genes of interest, with 263 TF with at least one associated binding site. There were 16 statistically over-represented TFBS in the dataset, including CEBP, cETS1p54 (ETS-1), CP2, E47, ELK1, IK1, IK3, NFKappaB, NFKappaB50, NRF2, SREBP1, SRF, STAT, TAL1ALPHAE47, USF and YY1 (Fig 6). The identified overrepresented binding sites were associated with 38–90% of all the 39 analysis-ready genes. USF (731 sites) and ELK1 (349 sites) had the most associated binding sites (Table 2). Only 11 of the 30 genes (36%) identified in cluster 10 were deemed analysis ready, mouse orthologs were not identified for 17 genes and TFBS were not found for 2 genes. There were only 2 statistically over-represented TFBS in this cluster, BRN2 and HFH1. Due to the lack of identified genes and associated TFBS, this cluster was excluded for all remaining analysis and focused all remaining analysis on Cluster 4.

**Table 2:** Overrepresented TFBS and associated genes with sites.

Transcription Factor Binding Site(s)	# Site(s)	# Genes with Site(s)	% Genes with Site(s)	Genes with Site(s)
CEBP	122	25	64	SALL3, TMEM86B, EVC, SCAMP2, AMDHD2, KCNH6, MAPKAPK3, PAK4, PLCD3, ABCB9, JPH3, KCNH3, SYT7, FNDC5, HR, TAX1BP3, LHX3, SLC2A10, COL5A2, PHOSPHO1, ZC3H7B, OPA, UNC119, MBD6, IRS1
CETS1P54	235	28	72	SALL3, TMEM86B, EVC, SCAMP2, AMDHD2, KCNH6, MAPKAPK3, PAK4, CRADD, PLCD3, ABCB9, JPH3, KCNH3, SYT7, FNDC5, INPPL1, HR, TAX1BP3, SLC2A10, COL5A2, PHOSPHO1, SLC5A6, OPA3, CHRNB3, UNC119, MBD6, PTRH1, IRS1
CP2	287	31	79	SALL3, TMEM86B, EVC, SCAMP2, AMDHD2, KCNH6, MAPKAPK3, PAK4, PLCD3, ABCB9, JPH3, KCNH3, SYT7, FNDC5, INPPL1, HR, TAX1BP3, LHX3, SLC2A10, COL5A2, PHOSPHO1, ZC3H7B, SLC5A6, EMP3, OPA3, UNC119, TCHHL1, SRMS, MBD6, PTRH1, IRS1
E47	176	25	64	SALL3, TMEM86B, SCAMP2, AMDHD2, CXCL14, KCNH6, PAK4, CRADD, PLCD3, JPH3, KCNH3, SYT7, FNDC5, INPPL1, HR, TAX1BP3, ZC3H7B, SLC5A6, GLIS2, UNC119, TCHHL1, SRMS, MBD6, PTRH1, IRS1
ELK1	349	31	79	SALL3, TMEM86B, EVC, SCAMP2, AMDHD2, KCNH6, B3GNT6, MAPKAPK3, PAK4, CRADD, PLCD3, ABCB9, JPH3, KCNH3, SYT7, FNDC5, INPPL1, HR, TAX1BP3, LHX3, SLC2A10, COL5A2, PHOSPHO1, POLM, ZC3H7B, EMP3, OPA3, UNC119, TCHHL1, MBD6, IRS1
IK1	199	27	69	SALL3, TMEM86B, SCAMP2, AMDHD2, KCNH6, MAPKAPK3, PAK4, CRADD, PLCD3, ABCB9, JPH3, KCNH3, SYT7, FNDC5, INPPL1, HR, TAX1BP3, LHX3, COL5A2, PHOSPHO1, POLM, EMP3, OPA3, UNC119, TCHHL1, MBD6, IRS1
IK3	109	25	64	SALL3, TMEM86B, SCAMP2, AMDHD2, KCNH6, MAPKAPK3, PAK4, CRADD, ABCB9, JPH3, KCNH3, SYT7, FNDC5, HR, TAX1BP3, LHX3, COL5A2, PHOSPHO1, POLM, ZC3H7B, EMP3, OPA3, UNC119, MBD6, IRS1
NFKAPPAB	150	30	77	SALL3, TMEM86B, EVC, SCAMP2, AMDHD2, CXCL14, KCNH6, PAK4, ABCB9, JPH3, KCNH3 SYT7, FNDC5, INPPL1, HR, TAX1BP3, LHX3, COL5A2, PHOSPHO1, ZC3H7B, SLC5A6, EMP3, OPA3, CHRNB3, UNC119, TCHHL1, SRMS, MBD6, PTRH1, IRS1
NFKAPPAB50	37	15	38	SALL3, TMEM86B, EVC, SCAMP2, PAK4, ABCB9, JPH3, KCNH3, SYT7, FNDC5, HR, PHOSPHO1, EMP3, MBD6, IRS1
NRF2	162	31	79	SALL3, TMEM86B, EVC, SCAMP2, AMDHD2, KCNH6, PAK4, CRADD, PLCD3, ABCB9, JPH3, KCNH3, SYT7, FNDC5, INPPL1, HR, TAX1BP3, SLC2A10, COL5A2, PHOSPHO1, POLM, ZC3H7B, SLC5A6, OPA3, CHRNB3, TAP2, UNC119, TCHHL1, MBD6, PTRH1, IRS1
SREBP1	107	26	67	SALL3, TMEM86B, EVC, SCAMP2, AMDHD2, CXCL14, KCNH6, MAPKAPK3, PAK4, CRADD, JPH3, KCNH3, SYT7, FNDC5, HR, COL5A2, PHOSPHO1, ZC3H7B, SLC5A6, OPA3, UNC119, TCHHL1, SRMS, MBD6, PTRH1, IRS1
SRF	66	19	49	SALL3, SCAMP2, AMDHD2, KCNH6, B3GNT6, PAK4, CRADD, PLCD3, SYT7, FNDC5, HR, TAX1BP3, COL5A2, ZC3H7B, SLC5A6, UNC119, MBD6, PTRH1, IRS1
STAT	67	23	59	SALL3, TMEM86B, EVC, SCAMP2, KCNH6, B3GNT6, PAK4, CRADD, ABCB9, JPH3, KCNH3, SYT7, FNDC5, HR, TAX1BP3, SLC2A10, COL5A2, PHOSPHO1, ZC3H7B, OPA3, UNC119, MBD6, IRS1
TAL1ALPHAE47	82	25	64	SALL3, TMEM86B, SCAMP2, AMDHD2, CXCL14, KCNH6, PAK4, CRADD, PLCD3, JPH3, KCNH3, SYT7, FNDC5, HR, SLC2A10, COL5A2, ZC3H7B, SLC5A6, GLIS2, UNC119, TCHHL1, SRMS, MBD6, PTRH1, IRS1
USF	731	35	90	SALL3, TMEM86B, EVC, SCAMP2, AMDHD2, CXCL14, KCNH6, B3GNT6, MAPKAPK3, PAK4, CRADD, PLCD3, ABCB9, JPH3, KCNH3, SYT7, FNDC5, INPPL1, HR, TAX1BP3, LHX3, SLC2A10, COL5A2, PHOSPHO1, ZC3H7B, SLC5A6, GLIS2, OPA3, TAP2, UNC119, TCHHL1, SRMS, MBD6, PTRH1, IRS1
YY1	61	21	54	SALL3, TMEM86B, SCAMP2, AMDHD2, KCNH6, PAK4, CRADD, PLCD3, JPH3, KCNH3, SYT7, INPPL1, HR, TAX1BP3, LHX3, COL5A2, SLC5A6, OPA3, UNC119, MBD6, IRS1

**Fig 7:**
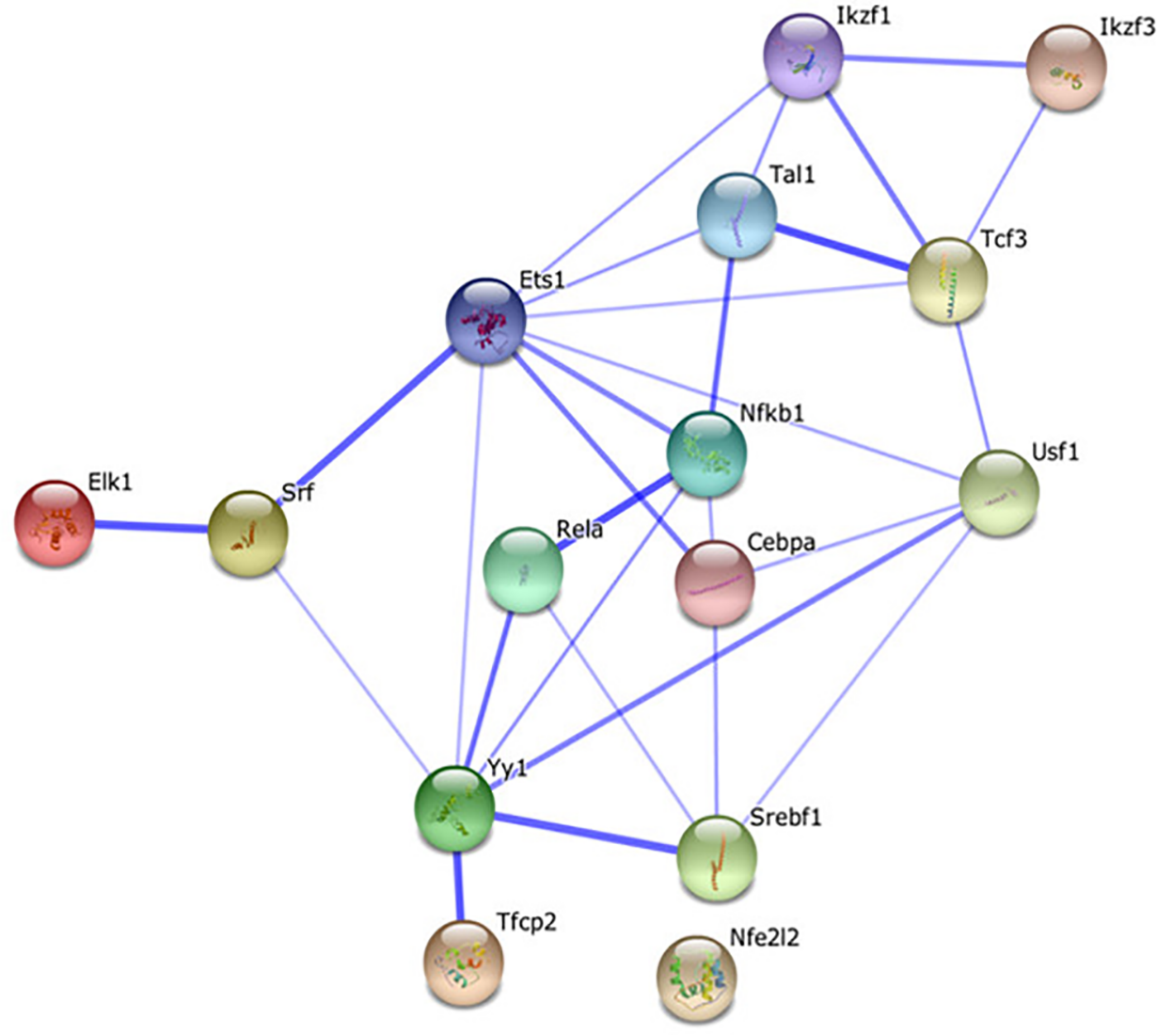
STRING database analysis. Protein—protein interactions between the over-represented TFBS from the CONFAC analysis. Thicker lines are a demonstration of stronger functional association. ETS- 1 had the most protein-protein interactions, with interactions with Srf, Yy1, Cebpa (CEBP), NfkB1 (NfKappaBp50), Tcf3 (E47), Tal1 (Tal1alpha47), Ikzf1 (IK1), and Usf1.

The STRING database was used to understand the relationships between the TF identified by CONFAC analysis. This database allows users to obtain a visualized network of protein-protein interactions from a list of proteins entered into a web-based user interface. The STRING database was able to identify 15 of the 16 over-represented CONFAC analysis TFBS and protein-protein interactions were identified among the associated TF. ETS-1 had the most interactions and was deemed to be the most central TF with eight direct interactions and connection to all proteins except Nrf2 (Nfe2l2) (Fig 7).

**Fig 8:**
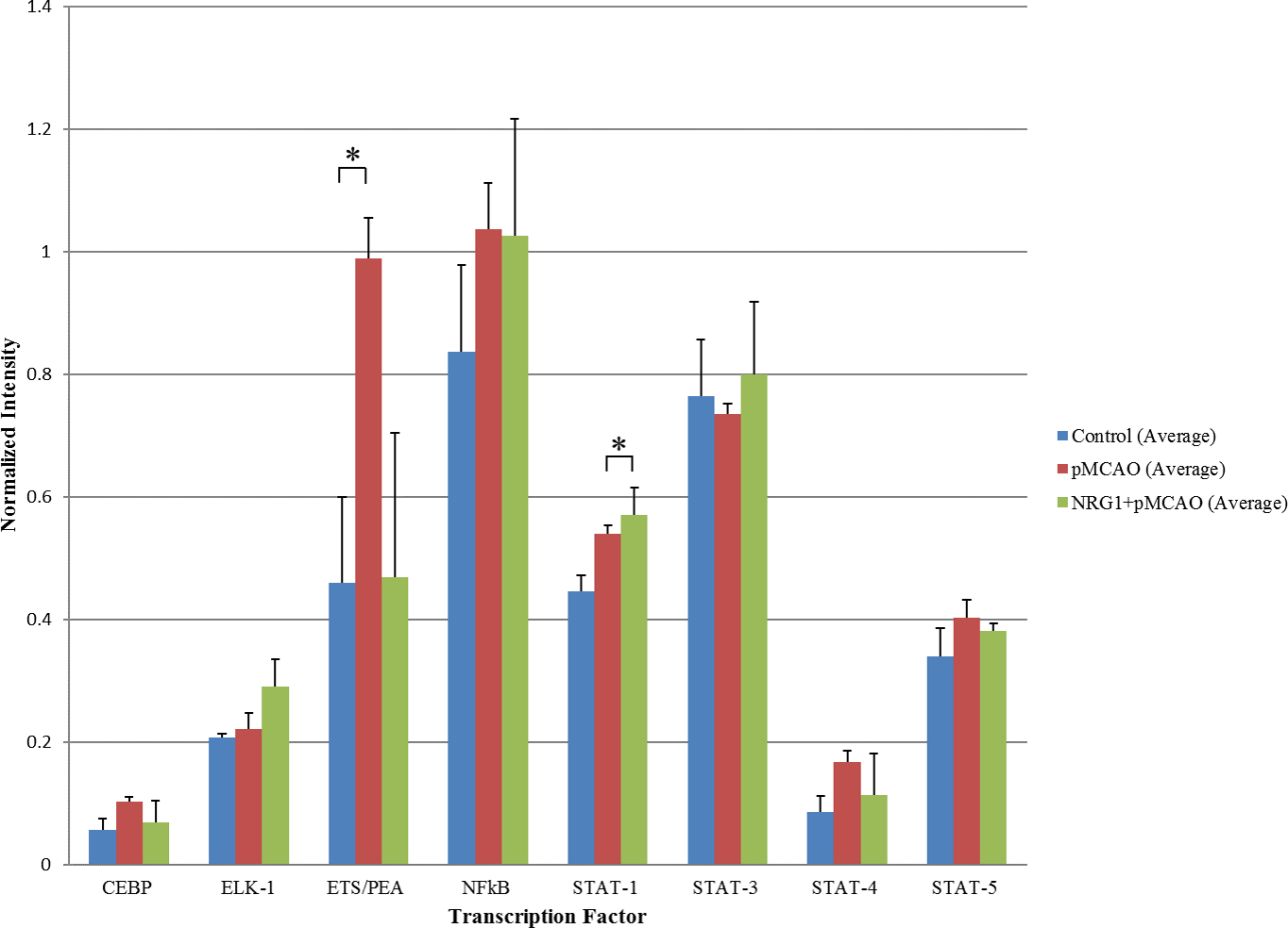
Transcription factor array. (a) STAT-1 and ETS-1 activity were increased 3 hours following ischemia and NRG1 treatment. NRG1 treatment reduced the activity of ETS-1 following stroke.

To examine whether the predicted transcriptional regulators were involved with ischemia and NRG1 neuroprotection, we conducted multiplex transcription factor assays on nuclear extracts from the brains of control, MCAO, and MCAO+NRG1 animals. Five of the 16 transcription factors identified by CONFAC analysis were present on the transcription factor array plate, which was CEBP, ELK-1, ETS/PEA (ETS-1), NFκB and STAT (STAT-1, STAT-3, and STAT-5) (Fig 8). ETS-1 (p = 0.04) and STAT1 (p = 0.05) were the only TF that showed a statistical difference between treatment groups at 3 hours post-ischemia. ETS-1 activity increased by 2-fold at 3 hours following ischemia, which was blocked by NRG1.

## Discussion

Following ischemia in the rat brain, neuronal damage begins early in the striatum, then spreads to the cortical areas (the penumbra) in the hours after ischemia [27]. In the present study, we used microarray technology to examine the molecular events occurring early in the cortex following MCAO model to determine how NRG1 treatment affects the propagation of ischemic damage to the penumbra. There was heavy FJB labeling in the striatum, but limited FJB staining in the cortex following MCAO. This indicates that at this early time point the injury may not have progressed to irreversible neuronal damage, which is indicative of the ischemic penumbra [44]. This study also provides evidence that NRG1 may provide early neuroprotection in the growing ischemic penumbra. Bioinformatic analysis identified clusters of genes that exhibit specific expression patterns where genes were altered following MCAO, but returned towards control levels following treatment with NRG1. We used pathway analysis tools, IPA and Enrichr, to understand the biological mechanisms associated with these clusters/genes of interest. CONFAC analysis was used to predict TF that could regulate these genes based on overrepresented TFBS associated with this set of genes.

Although NRG1 was not present in the cluster of genes analyzed, a network analysis revealed that NRG1 was a common link to many of the genes in this cluster. IPA curated references identified genes within cluster 4 namely, OPA3, IRS1, CHRNB3, and POU6F1, that have been previously associated with NRG1 [45–48]. The transcription factor POU6F1 (Brn-5) has been shown to be regulated by NRG1 and is involved in Schwann cell myelination [47]. Stress-dependent transactivation of NRG1 receptors was shown to induce phosphorylation of IRS proteins resulting in insulin resistance [45]. IRS-1 has also been linked to ischemic stroke, possibly being an indicator of cardiovascular disease and increasing insulin resistance [49, 50].

Many of the transcription factors identified in this study have previously been associated with mechanisms related to ischemic stroke. The most notable were NFκB [51, 52] and STAT [53–55], which have both been associated with regulating inflammatory mechanisms leading to cell death following ischemic stroke. CEBP has also been identified in microglia cells following ischemic stroke and has been shown to mediate ischemic neuronal damage [56, 57]. The expression levels of SRF [58], ELK1 [59], SREBP1 [60], and NRF-2 [61] have been shown to increase in ischemic stroke models. Also, we previously showed that the transcription factor IK1 was associated with genes that increased following transient MCAO using CONFAC [29]. CP2, E47, IK3, TAL1, USF, and YY1 are TF that has not been previously shown to be associated with stroke. However, YY1 has been shown to mediate the effects of NRG1 on transcriptional modulation of peripheral myelination by Schwann cells [62]. Several of these transcription factors have previously been linked to neurodegeneration [63–66], which may provide additional information about the mechanisms through which NRG1 regulates ischemia-induced genes.

A key transcriptional regulator of the genes analyzed by CONFAC was ETS-1. ETS-1 (v-Ets Avian Erythroblastosis Virus E26 Oncogene Homolog 1) was discovered from an avian erythroblast virus (E26) myb-ets fusion oncogene. ETS-1 was the first identified protein in the ETS family of transcription factors, consisting of 12 subfamilies of proteins, including ETS, ELF, ELG, ERG, ERF, ESE, PSEF, PEA3, ER71, SPI, TCF and TEL [67–69]. ETS-1 has been previously associated with many biological processes, including embryonic development and inflammation [67–69]. ETS-1 requires either protein-protein interactions with other transcription factors, such as Runx1, Pax5, USF1, STAT5, and TFE or phosphorylation of the threonine residue (Thr38). Phosphorylation of the serine residues in the auto-inhibitory segment of ETS-1 has been shown to prevent binding of DNA [70], which may be relieved by interactions with several proteins, including USF1 [71], which was also identified in the CONFAC analysis. ETS-1 has been previously shown to bind directly to and increase IKKα expression, which is associated with activation of the NFκB alternative pathway and inhibition of the NFκB canonical pathway mediated pro-inflammatory mechanisms [70–72]. Using CONFAC, we previously showed that ETS-1 was associated with neuronal death and inflammation in a rat stroke model [29]. Enrichr, STRING and transcription factor assay analyses all indicated that ETS-1 may play a role in the neuroprotective and anti-inflammatory effects of NRG1 in the brain following MCAO. ETS-1 family members have been shown to be involved in NRG1 mediated Schwann cell survival [72]. Studies are underway to understand the mechanisms by which ETS-1 is regulated following ischemic stroke and how it is affected by NRG1.

## Conclusion

NRG1 is neuroprotective following ischemic stroke. ETS-1 was identified as a candidate transcription factor associated with the regulation of gene expression by stroke and NRG1 treatment. NRG1 is currently in a phase III human clinical trial for heart failure and showed significant efficacy for improving cardiac function human patients [73, 74]. Therefore, understanding the mechanisms used by NRG1 to prevent neuronal cell death could lead to the development of a novel strategy to treat stroke.

## Supporting information

S1 Table Genes located in k-means clusters. (XLSX)
